# Epitope vaccine design for *Toxoplasma gondii* based on a genome-wide database of membrane proteins

**DOI:** 10.1186/s13071-022-05497-z

**Published:** 2022-10-12

**Authors:** Xuan-Wu Li, Ni Zhang, Zhuo-Lin Li, Nouhoum Dibo, Zhen-Rong Ma, Bin Lu, Ye-Hong Huang, Yun-Feng Chang, Hong-Zhi Chen, Xiang Wu

**Affiliations:** 1 National Clinical Research Center for Metabolic Disease, Key Laboratory of Diabetes Immunology, Ministry of Education, and Department of Metabolism and Endocrinology, The Second Xiangya Hospital of Central South University, Changsha, China; 2 Department of Parasitology, Xiangya School of Basic Medicine, Central South University, Changsha, China; 3 Hunan Provincial Key Lab of Immunology and Transmission Control on Schistosomiasis, Changsha, China; 4 Department of Forensic Medicine Science, Xiangya School of Basic Medicine, Central South University, Changsha, China

**Keywords:** *Toxoplasma gondii*, Epitope vaccine, Bioinformatics, Human leukocyte antigen

## Abstract

**Background:**

There is presently no effective and safe vaccine for *Toxoplasma gondii* for humans. The study described here was designed to search for a novel group of optimal B cell and T cell epitopes from *Toxoplasma* membrane proteins using genome-wide comprehensive screening.

**Methods:**

The amino acid sequences of membrane proteins of *T. gondii* were obtained from the UniProt database. The ABCPred and BepiPred servers were employed to predict the linear B cell epitopes. The Immune Epitope Database (IEDB) online service was utilized to forecast T cell epitopes within *T. gondii* membrane proteins that bind to human leukocyte antigen (HLA) class I (HLA-I) or HLA-II molecules.

**Results:**

From the 314 membrane proteins of *T. gondii*, a total of 14 linear B cell epitopes embedded in 12 membrane proteins were identified. Eight epitopes for major histocompatibility complex (MHC) class I (MHC-I) molecules and 18 epitopes for MHC-II molecules were ultimately selected, for which world population coverage percentiles were 71.94% and 99.76%, respectively. The top rated combinations of linear B cell epitopes and T cell epitopes covering both BALB/c mice and a majority of the human population were identified for the development of a protective vaccine.

**Conclusions:**

The ultimate vaccine construct described here, which comprises B cells, MHC-I and MHC-II epitopes, might protect individuals against *T. gondii* infection by inducing humoral and cellular immune responses.

**Graphic abstract:**

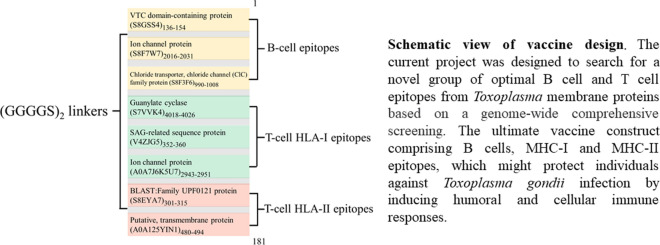

**Supplementary Information:**

The online version contains supplementary material and is available at 10.1186/s13071-022-05497-z.

## Background

*Toxoplasma gondii* is an intracellular parasite that causes toxoplasmosis in humans and animals [1, 2]. Infection with this parasite during pregnancy can lead to infection of the fetus, and may cause miscarriage or stillbirth [3]. Moreover, this infection can be lethal in immunocompromised individuals. Toxoplasmosis is also prevalent among domestic animals, and may result in abortion, especially in goats and sheep, which can cause enormous losses to the livestock industry [4]. Thus, protecting humans and livestock from *Toxoplasma* infection is important for public health.

Vaccination is a more effective approach for *T. gondii* than chemotherapy [5]. However, there is as yet no effective and safe vaccine for *T. gondii* for humans, mainly due to limitations in vaccine development strategies and lack of good candidate vaccine molecules [5]. Most of the current studies on epitopes only focus on some characterized proteins of *T. gondii*, and there has been no extensive and systematic search of *Toxoplasma* proteins. Moreover, experimental results derived from mice, the most widely used animal model for *T. gondii* vaccine development, are inevitably biologically biased due to the genetic differences between mice and humans [6].

**Fig. 1:**
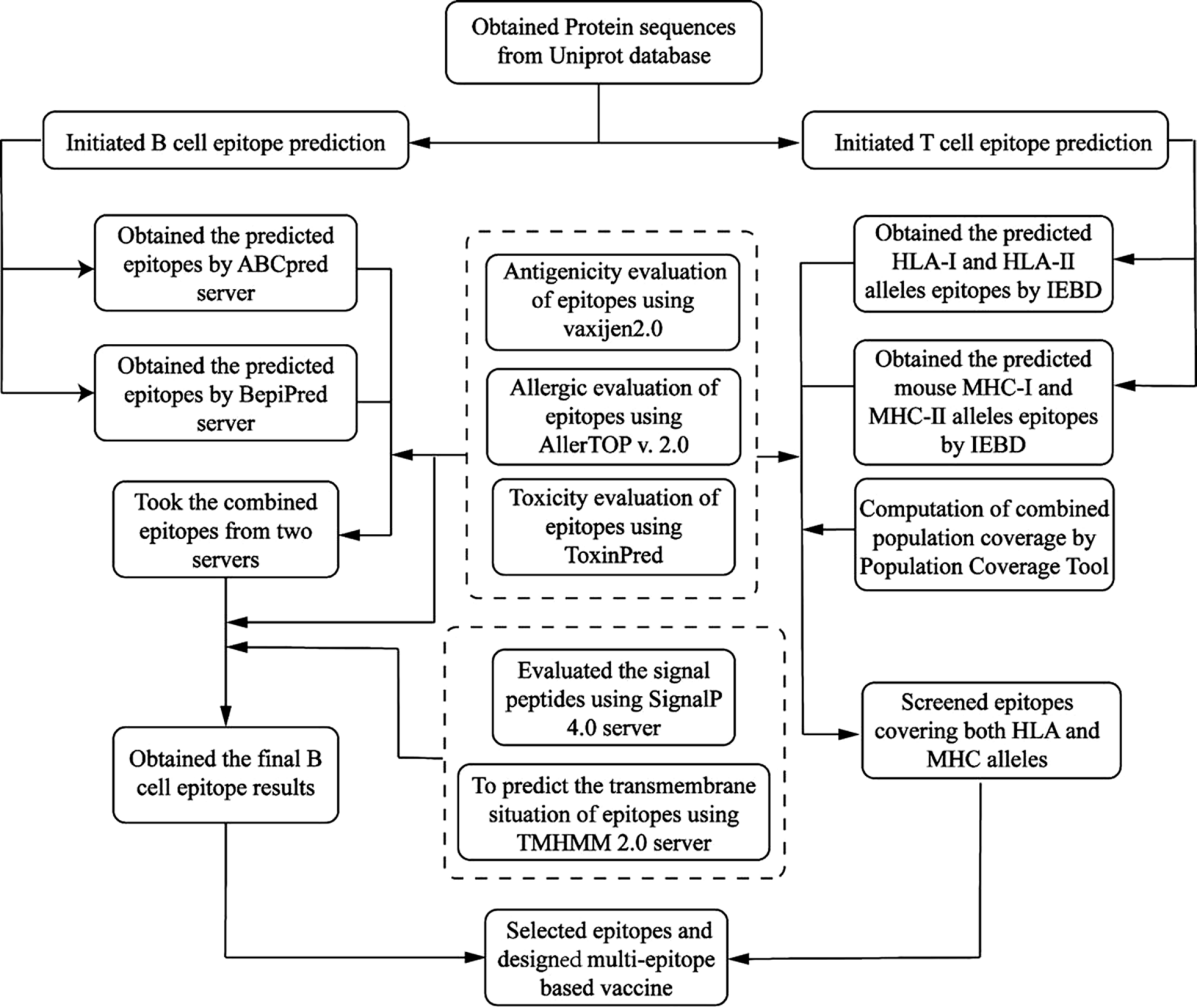
Workflow diagram

The present study was designed to unbiasedly search for a novel group of optimal B cell and T cell epitopes. Bioinformatics and immunoinformatic analysis were conducted for a genome-wide range of *Toxoplasma* membrane proteins (including uncharacterized proteins). The steps of the in silico analysis are illustrated in Fig. 1.

## Methods

### Linear B cell epitope prediction

Surface proteins and cell membrane proteins were identified as targets. The amino acid sequences of membrane proteins of *T. gondii* were obtained from the UniProt database (https://www.uniprot.org/uniprot/). ABCPred [7], and the BepiPred [8] servers were employed to predict the linear B cell epitopes. The window length of the ABCPred server prediction results was set to 16, and the threshold value adjusted to 0.95 due to the large number of predictions obtained. For BepiPred server 2.0, the threshold value was set to 1.5 to screen for epitopes with high scores. When the results of the ABCPred and BepiPred servers converged, we used the combined results as the candidate linear B cell epitopes for the subsequent analysis.

###  Prediction of mutual epitopes of human leukocyte antigens and mouse major histocompatibility complex

The Immune Epitope Database (IEDB) online service was utilized to forecast T cell epitopes within *T. gondii* membrane proteins that bind to human leukocyte antigen (HLA) class I (HLA-I) or HLA-II molecules [9, 10]. For HLA-I molecules, the HLA allele reference set was selected and peptides with assessment scores greater than 0.7 were selected in the screening. For HLA-II molecules, the full HLA reference set was selected and the peptides in the top 0.5% were selected as the screening range for the prediction results. Computation of the combined population coverage was conducted using the Population Coverage tool [11].

BALB/c mice, the laboratory animals most commonly used in vaccine studies, were selected as the targets to screen for major histocompatibility complex (MHC) molecules that bind to epitopes. Three MHC class I (MHC-I) alleles (*H2-Db*,* H2-Kb* and* H2-Ld*) and two MHC-II alleles (*H2-IAd* and* H2-IEd*) from BALB/c mice were employed [9, 10, 12]. We retained the MHC-I epitopes with scores greater than 0.7 and the MHC-II epitopes that were ranked in the top 0.5%. The epitopes’ binding abilities for each allele were evaluated and listed.

### Profiling and evaluation of predicted epitopes

Vaxijen2.0 server [13], AllerTOP v. 2.0 [14] and ToxinPred [15] were employed to evaluate the antigenicity, allergenicity and toxicity of all epitopes; these tools were applied using their default settings. The SignalP 5.0 server was used [16] to exclude the epitopes which embody signal peptides. The TMHMM 2.0 server [17] was utilized to analyze the transmembrane situation of epitope-derived proteins. Epitopes located in the outer membrane at a probability greater than 0.85 were recruited. ToxinPred was employed to screen for epitopes with good hydrophilicity.

## Results and discussion

Over a third of the global human population is in danger of *T. gondii* infection. The currently available anti-toxoplasmosis vaccines, including live attenuated vaccines, lysis products and excreted/secreted antigen vaccines, are all for non-human use [18]. Live attenuated vaccines emulated natural infection and provided a microenvironment for antigen processing and presentation similar to that of *T. gondii* infection [18–20]. A commercial vaccine, Toxovax, based on the live attenuated tachyzoites of the S48 strain of* T. gondii*, has been licensed for use in New Zealand, the UK and some European countries to reduce the incidence of abortion in sheep that results from infection [21]. Most of the current anti-toxoplasmosis vaccines were developed by targeting a number of protein families such as surface antigens, rhoptry bulb proteins, microneme proteins, and dense granule proteins as antigens [18–20, 22].

Currently, antigen selection is mostly based on proteins identified without an extensive search of the *Toxoplasma* gene database. Large-scale screening would enhance the likelihood of identifying potentially promising epitopes. In order to select the best epitopes, we targeted membrane proteins of *T. gondii* from the UniProt database, and obtained 314 of them. Proteins without an extracellular domain were excluded from the following. Information on the proteins is presented in Additional file 1 and their amino acid sequences in Additional file 2. A total of 238 epitopes were predicted by the ABCPred server with the threshold value set to 0.95 (Additional file 3). Of these predicted epitopes, 32 peptides were also identified by BepiPred 2.0 server (Additional file 15: Table S1). Fourteen linear B cell epitopes with high scores of antigenicity computed via vaxijen2.0 were selected. These 14 B cell epitopes of *T. gondii* were distributed on 12 membrane proteins (Table 1); their immunobiological characteristics are presented in Additional file 16: Table S2. The overlapping parts of the epitopes identified by both the ABCPred and BepiPred-2.0 servers are shown in Additional file 11: Figure S1. The distribution of these epitopes on the corresponding proteins is shown in Additional file 12: Figure S2. All of these epitopes have probabilities > 0.85 of being exposed towards the outside of cells.

To the best of our knowledge, this work is the first systematic epitope vaccine screening of all *T. gondii* membrane proteins through bioinformatics analysis. Within this wide range of target proteins, the retained B cell and T cell epitopes were non-allergenic, non-toxic, and had good antigenicity. For B cell epitopes, we also proceeded with the prediction of signal peptides, hydrophilicity and transmembrane motifs in the proteins.

**Table 1:** Summary of the selected linear B cell epitopes and corresponding proteins

Entry	Protein name	Peptide	Position	Length
S8EQX7	Carrier protein superfamily	DAPSDGEGSGPRQGDREVTT	520–539	20
S8FBJ7	Membrane protein, putative	GGRQIENGGEDAAVND	936–951	16
Q6GV23	Rhomboid-like protease 5	EQPPTGDYKRRALASP	51–66	16
S8GSS4	Vacuolar transporter chaperone (VTC) domain-containing protein	VGSQESGQARERDDREATE	136–154	19
S8GRC2	Uncharacterized protein [Basic Local Alignment Search Tool (BLAST): organic solute transporter ostalpha protein]	DGRREASEDPSVSANPHPTDSARTTSPSADDQ	58–89	32
		SAPGQEPQSPRGNADPRPSG	1015–1034	20
S7WII9	CDC50-related protein CDC50.1	YQFLEGTPEGDNDQVP	317–332	16
S8EV89	Uncharacterized protein (BLAST: putative transmembrane protein)	EREETAREEWREDSTPRRS	583–601	19
S7UQV8	Unique GC organizer (UGO)	DSRWGSGSEASADAYP	1430–1445	16
S8F7W7	Ion channel protein	WESWGVPTDPESRANE	2016–2031	16
S8F3F6	Chloride transporter, chloride channel (ClC) family protein	AEGEGRGDSRDSRDVRLCT	990–1008	19
		MPGRGEGESSGDEIEETRDHDVGCFD	1124–1149	26
A0A125YIN1	Uncharacterized protein (BLAST: putative transmembrane protein)	GVPGQLPRTRGPYTSP	65–80	16
S8GDU1	Cleft lip and palate transmembrane protein 1 (Clptm1)	VPGGSSGGGPAGDAQAPP	7–24	18

The population coverage of the HLA alleles was predicted by the Population Coverage tool of IEDB Analysis Resource (http://tools.iedb.org/population/). The global population coverage rate as well as those of 11 major regions of the world were computed. For HLA-I, nine world regions showed more than 50% population coverage, with the highest population coverage in Europe, at 81.48%, and in North America, at 76.79% (Additional file 13: Figure S3a). For HLA-II, the population coverage rates of all 11 world regions exceeded 95% (Additional file 13: Figure S3b). T cell epitope screening of the 314 membrane proteins of *T. gondii* was conducted using the IEDB online service. For HLA-I binding epitopes, the selected scores ranged from > 0.7, and we obtained 1221 initial predictions (Additional file 4). For HLA-II binding epitopes, results with prediction scores that were ranked in the top 0.5% were selected, which gave 19,241 initial epitopes candidates (Additional file 5).

Because of the inherent genetic differences between mice and humans, conclusions based on the results of experiments using mice cannot be directly extrapolated to humans. For example, immunity-related guanosine triphosphatases assist mice during early *Toxoplasma* infection, but humans lack most of the genes in this protein family [23]. Nevertheless, in vivo experimental validation using mice is still valuable and important. In sum, the designed epitopes should ideally be recognized by both HLA and mouse MHC. The T-cell epitope prediction using mouse MHC molecules was conducted online, and resulted in 920 mouse MHC-I binding epitopes (*H2-Db*,* H2-Kb* and* H2-Ld*) with a score > 0.7 (Additional file 6) and 1185 mouse MHC-II binding epitopes (H2-IAd, H2-IEd] that were ranked in the top 0.5% (Additional file 7). Subsequently, we identified 909 epitopes capable of binding to both HLA-I and mouse MHC-I molecules, and 526 epitopes capable of binding to both HLA-II and mouse MHC-II molecules (Additional file 8). The epitopes with the highest score or highest rank were selected. After screening for antigenicity, allergenicity and toxicity, eight MHC-I binding epitopes were retained; these could be recognized by 12 HLA-I alleles and three mouse MHC-I alleles (Table 2). For MHC-II binding peptides, we selected 18 epitopes that can bind to 19 HLA-II alleles and two mouse MHC-II alleles (Table 2). The alleles covered and the corresponding scores or ranks of each epitope are shown in Additional file 14: Figure S4. As the selected epitopes could be recognized by both human and mouse MHC molecules, our strategy may be a successful approach to translating mouse-derived experimental results to humans for future vaccine development.

**Table 2:** Human leukocyte antigen (*HLA*) I and HLA II allele-binding epitopes predicted by the Immune Epitope Database server

Entry	Protein name	Epitope	Antigenicity
HLA I			
S7VVK4	Guanylate cyclase	YLHENVTFL	0.5471
V4ZJG5	Surface antigen (SAG)-related sequence protein SRS36C	VVFTPFVSF	1.0384
A0A7J6K7H4	Cyclic nucleotide-binding domain-containing protein	FPSSFPASF	1.3959
A0A0F7VBP5	Rhoptry kinase family protein (ROP) 5 (incomplete catalytic triad)	STATFTHAL	0.5805
S8F0B0	Palmitoyltransferase	SPRPGGLVL	1.5566
A0A7J6K5U7	Ion channel protein	SPHLRLTAF	1.5632
S8GUI2	Membrane protein, putative	LPYPVYSSL	0.7028
A0A125YP19	SAG-related sequence SRS30C	SSNANEYTF	0.9631
HLA II			
S8EYA7	Uncharacterized protein (BLAST: family UPF0121 protein)	IPIFLYFHLFRIRYS	1.2381
A0A125YIN1	Uncharacterized protein (BLAST: putative transmembrane protein)	EDRLRLSAAALAAAR	0.841
S8F2A3	GpcrRhopsn4 domain-containing protein	EQYHALAVMAAAIYV	0.5944
S8G829	Uncharacterized protein [BLAST: magnesium transporter nuclear-interacting partner of anaplastic lymphoma kinase (NIPA) protein]	PGGSLRAASAAVSSE	0.8423
A0A125YHS8	Ion channel protein	NQDQAMAAAAAGAAE	0.8502
G4XMZ5	Rhoptry protein 7	DAARLYLTVRAVRLV	0.6796
V4Z7H9	Calcium-activated potassium channel (slowpoke)	NRLVTDFKMRRKLKF	0.6342
S8EUE6	DUF803 domain-containing protein	PLYQRFRRTRSTASD	1.179
S8GTD3	PQ loop repeat-containing protein	TLLLFWRFRSARDGR	1.4365
A0A7J6K5U7	Ion channel protein	TPWRRLFRRTKVARL	0.694
B6KEU8	Dense granule protein 3 (P30)	AYYIRKVLTYRRRVM	0.6837
Q9GSE9	p35 SAG	LFRTAVVAAMAAALI	0.6593
Q07828	Dense granule protein 5 (protein GRA 5) (p21)	SLLRLLKRRRRRAIQ	0.8673
A0A0B5L9J2	Rhoptry protein 8	AAALRFFRRFRRVRQ	0.6075
A0A125YKW6	Uncharacterized protein (BLAST: putative membrane protein)	FYISFFYVNRTRLAA	0.5507
S8GPK0	TRAM/LAG1/CLN8 homology domain (TLC) domain-containing protein	AQILVAQAASQAYSS	0.734
S7UQV8	UGO	SQLFHFRRARRRHRR	1.0901
		RWFFSQLFHFRRARR	0.8294

For other abbreviations, see Table 1

**Fig. 2a, b:**
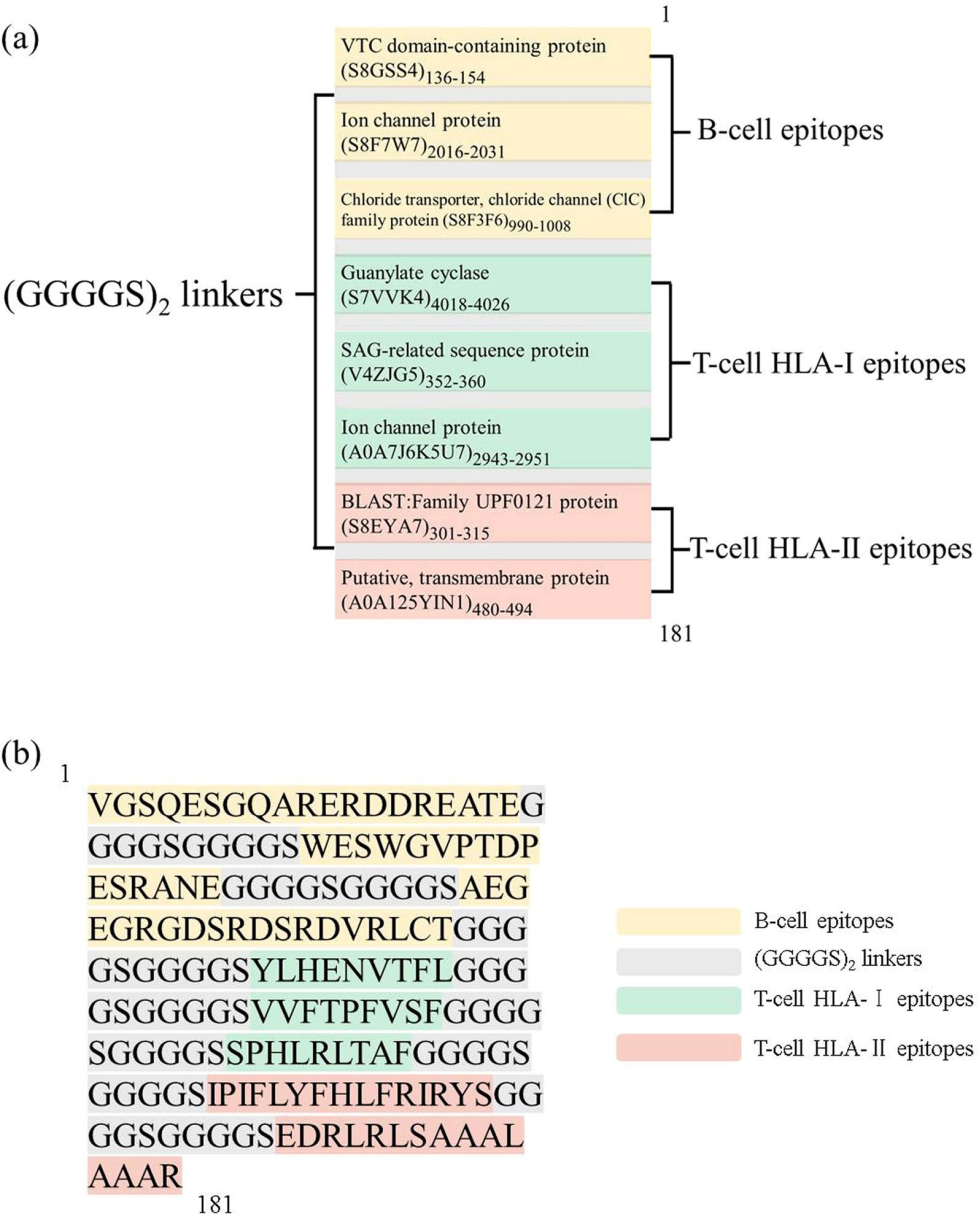
Epitope-based vaccine construct. **a** Schematic view of the vaccine design. **b** Amino acid sequence of the vaccine construct.

The tentative vaccine design contains three linear B cell epitopes, three MHC-I epitopes and two MHC-II epitopes (Fig. 2a). The B cell epitopes were chosen according to a holistic consideration of their antigenicity and hydrophilicity. For T cell epitopes, the selection was based on the corresponding score/rank and the population coverage. The identified epitopes all exhibit strong binding affinity to a series of MHC molecules (Additional file 9). The selected T cell epitopes could theoretically be recognized and presented by immune cells in more than 96% of the world’s population (Additional file 10). In addition, full coverage for three MHC-I and two MHC-II alleles in BALB/c mice indicates that there will also be a strong immune response in laboratory mice. The epitopes determined in this study were linked using (GGGGS)_2_ linkers for a chimeric antigen (Fig. 2b).

Despite the findings discussed above, the current study had several limitations. Firstly, the epitopes predicted and selected in this study need to be validated by in vitro or in vivo experiments. Secondly, both the efficacy and safety of the construct as an anti-*Toxoplasma* vaccine need to be tested. Thirdly, the current study focused on the epitope screening of *Toxoplasma* membrane proteins, but good epitope candidates may also exist that are located on cytosolic proteins.

Some of the epitopes identified here matched those from previous studies [18–20, 24]. In addition, some of the proteins from which they were identified are putative membrane proteins, and the T and B cell epitopes screened using them also exhibited excellent immunogenicity. Thus, these are potentially excellent candidate molecules. The search of the IEDB server showed that the (S8F3F6)_990–1008_ and (AOA125YINI)_480–494_ peptides in the vaccine construct, which are experimentally determined epitopes (Additional file 17: Table S3), harbor homologous sequences to yellow fever virus and *Yersinia pestis*, respectively. Admittedly, although in silico analysis is a powerful forecasting tool, its results are not substitutes for experimental evidence. Thus, the immunological characteristics of the epitopes identified here will be further evaluated in both in vitro and in vivo experiments.

## Supplementary Information

**Additional file 1.** Information on membrane proteins of *Toxoplasma gondii *obtained from the UniProt database.

**Additional file 2.** Amino acid sequences of the proteins presented in Additional file 1.

**Additional file 3**. Linear B cell epitopes predicted by the ABCPred server.

**Additional file 4.** Initial predicted HLA-I binding epitopes.

**Additional file 5.** Initial predicted HLA-II binding epitopes.

**Additional file 6.** Initial predicted mouse MHC-I binding epitopes.

**Additional file 7**. Initial predicted mouse MHC-II binding epitopes.

**Additional file 8.** Identified epitopes capable of binding to both HLA-I and mouse MHC-I molecules, and epitopes capable of binding to both HLA-II and mouse MHC-II molecules.

**Additional file 9. **Binding capacity of selected T cell epitopes.

**Additional file 10.** Worldwide population coverage of HLA alleles by the vaccine.

**Additional file 11: Figure S1.** Selected linear B cell epitopes and their positions on the corresponding proteins.

**Additional file 12: Figure S2.** Transmembrane motif analysis of proteins pertaining selected linear B cell epitopes. (Arrows and numbers indicate the positions of the epitopes in the protein sequences).

**Additional file 13: Figure S3.** Population coverage analysis. (a) Population coverage analysis of the 13 Recommended HLA-I alleles; (b) Population coverage analysis of the 19 Recommended HLA-II alleles

**Additional file 14: Figure S4.** Selected T cell binding epitopes. (a) CD8^+^ T cell epitopes and their corresponding MHC-I alleles in mice and human; (b) CD4^+^ T cell epitopes and their corresponding MHC-II alleles in mice and human

**Additional file 15: Table S1. **The initial result of the joint prediction using BepiPred-2.0 and ABCPred.

**Additional file 16: Table S2 **Antigenicity, allergenicity, toxicity, transmembrane localization, signal peptide and hydrophilicity profiling of selected peptides.

**Additional file 17: Table S3 **Alignment of experimentally verified epitopes and selected sequences in the vaccine construct.

## Data Availability

Not applicable.

## Abbreviations

HLA: Human leukocyte antigen
IEDB: Immune Epitope Database
MHC: Major histocompatibility complex
