# Supplementary figures and images for: Epitope vaccine design for Toxoplasma gondii based on a genome-wide database of membrane proteins
Source: Parasit Vectors. 2022 Oct 12;15:364. doi: 10.1186/s13071-022-05497-z (PMC9555269; doi:10.1186/s13071-022-05497-z)

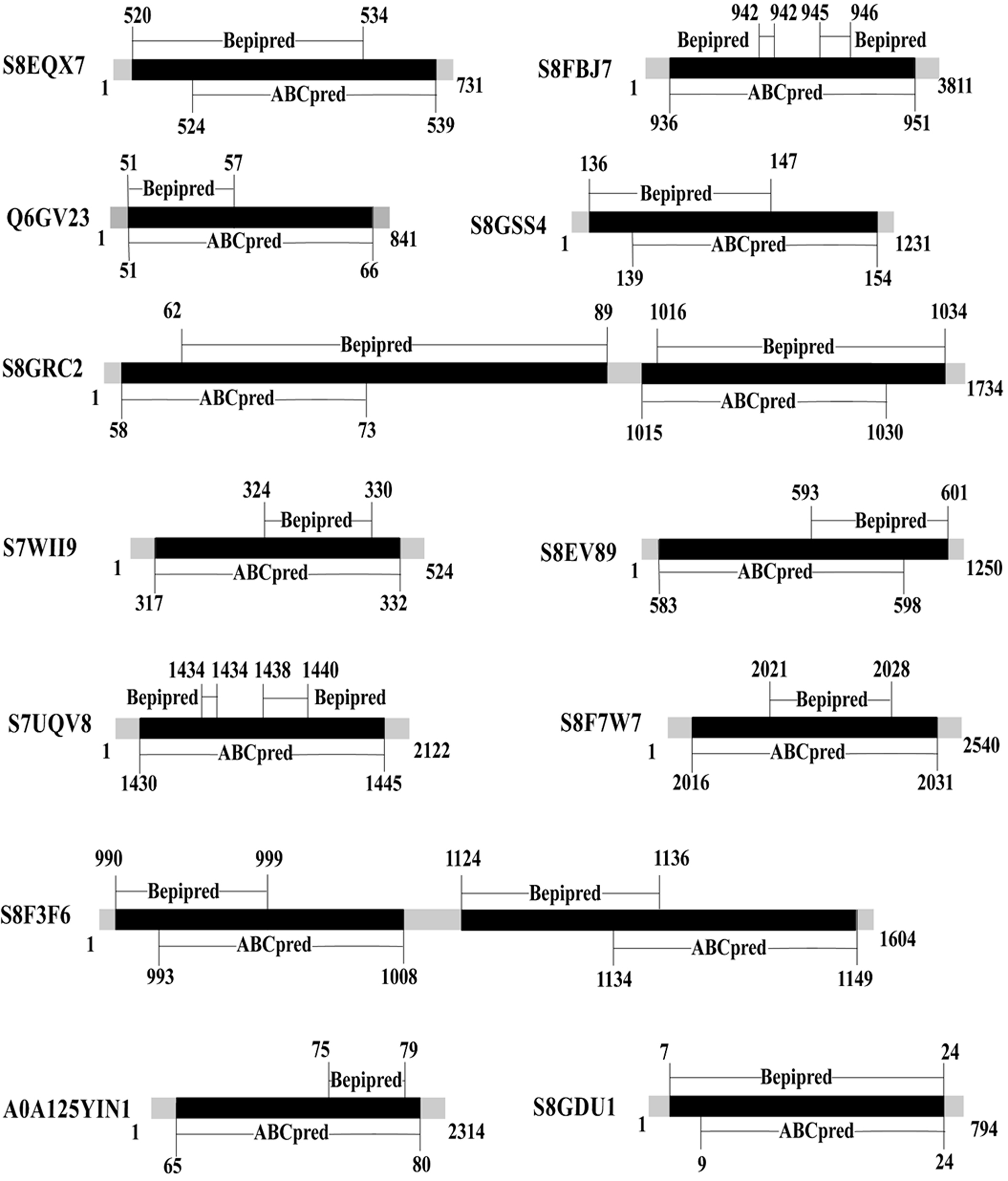

Supplement: Supplementary file 11 — Additional file 11: Figure S1. Selected linear B cell epitopes and their positions on the corresponding proteins. [file 13071_2022_5497_MOESM11_ESM.jpg]

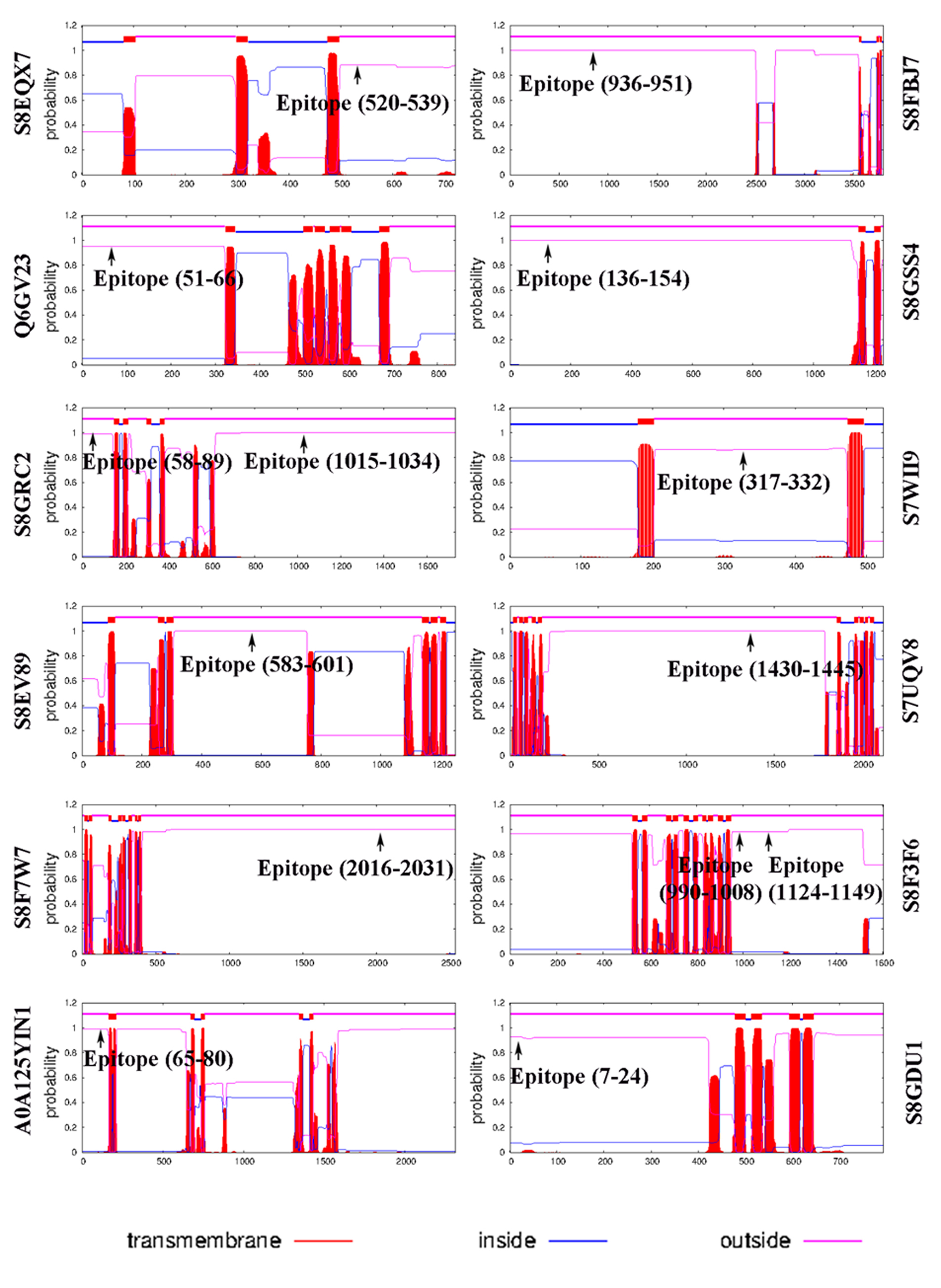

Supplement: Supplementary file 12 — Additional file 12: Figure S2. Transmembrane motif analysis of proteins pertaining selected linear B cell epitopes. (Arrows and numbers indicate the positions of the epitopes in the protein sequences). [file 13071_2022_5497_MOESM12_ESM.jpg]

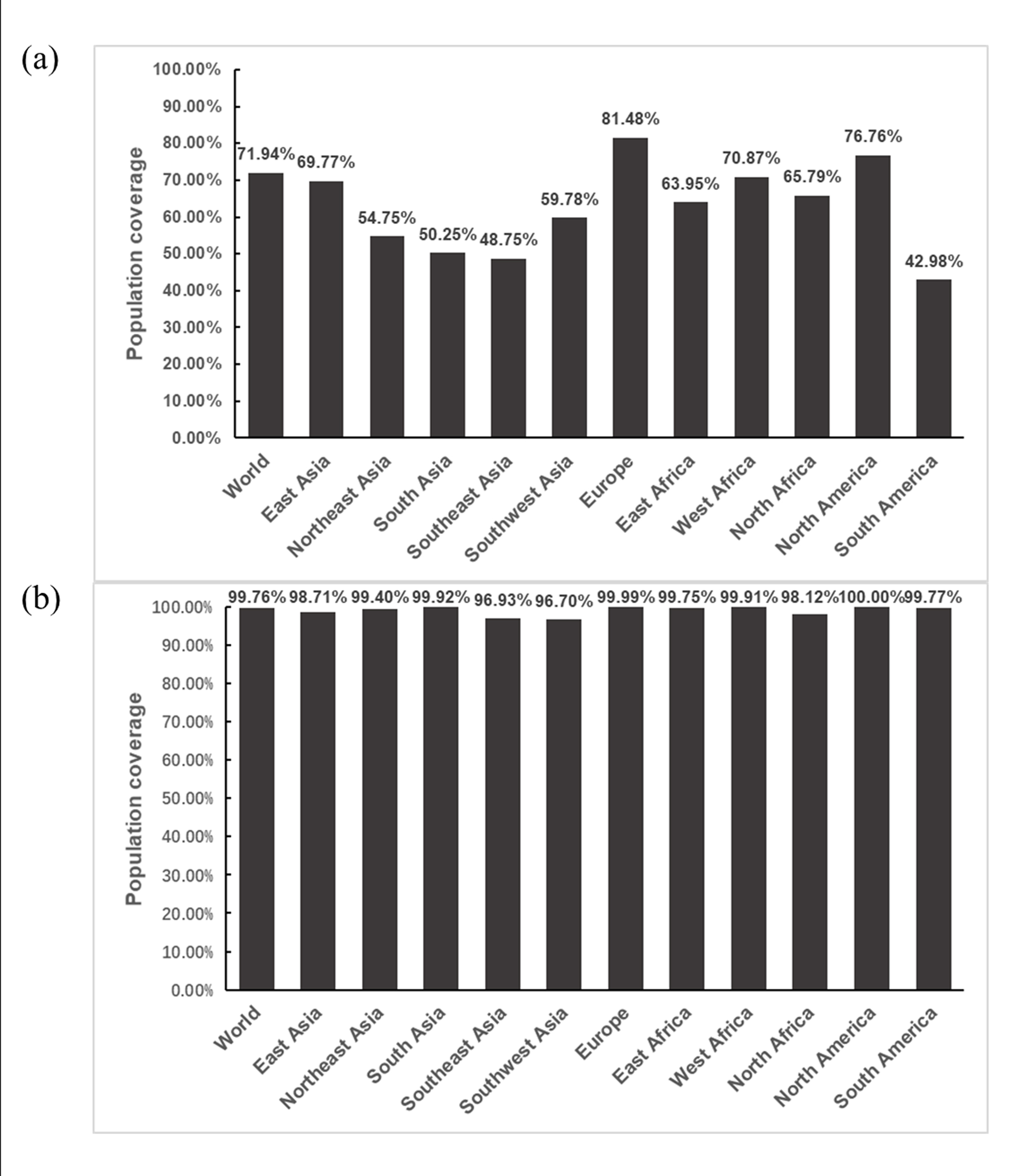

Supplement: Supplementary file 13 — Additional file 13: Figure S3. Population coverage analysis. (a) Population coverage analysis of the 13 Recommended HLA-I alleles; (b) Population coverage analysis of the 19 Recommended HLA-II alleles [file 13071_2022_5497_MOESM13_ESM.jpg]

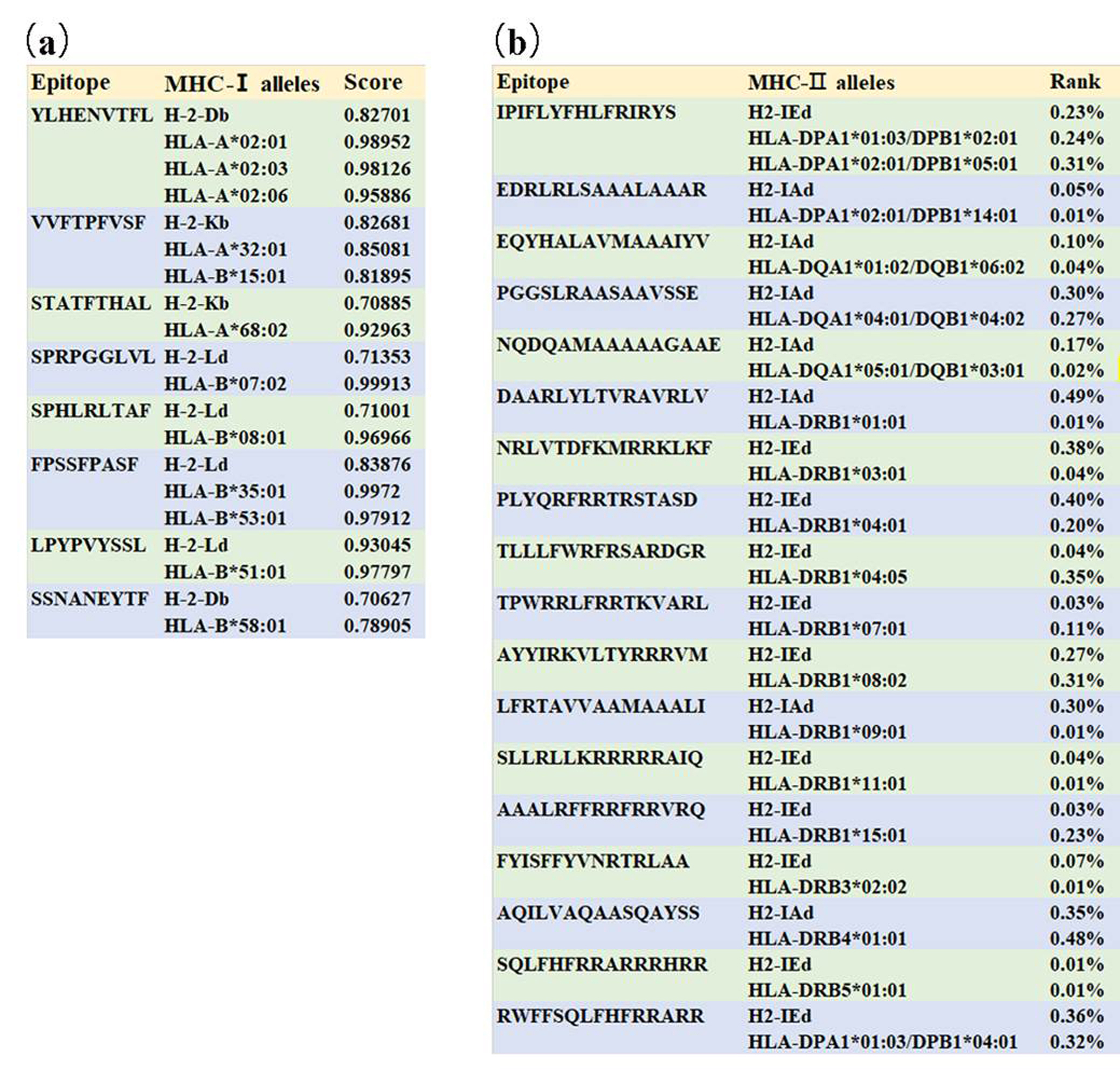

Supplement: Supplementary file 14 — Additional file 14: Figure S4. Selected T cell binding epitopes. (a) CD8+ T cell epitopes and their corresponding MHC-I alleles in mice and human; (b) CD4+ T cell epitopes and their corresponding MHC-II alleles in mice and human [file 13071_2022_5497_MOESM14_ESM.jpg]
